# The Influence of Using Recycled Waste Aggregates and Adding TiO_2_ Nanoparticles on the Corrosion Resistance of Steel Reinforcement Embedded in Cementitious Composite

**DOI:** 10.3390/ma17163895

**Published:** 2024-08-06

**Authors:** Carmen Teodora Florean, Mihail Chira, Horațiu Vermeșan, Timea Gabor, Andreea Hegyi, Claudia Alice Crișan, Cristina Câmpian

**Affiliations:** 1 Faculty of Materials and Environmental Engineering, Technical University of Cluj-Napoca, 103-105 Muncii Boulevard, 400641 Cluj-Napoca, Romania; carmen.florean@incerc-cluj.ro (C.T.F.); andreea.hegyi@incerc-cluj.ro (A.H.); claudia.crisan@imadd.utcluj.ro (C.A.C.); 2 National Institute for Research and Development in Construction, Urban Planning and Sustainable Spatial Development URBAN-INCERC Cluj-Napoca Branch, 117 Calea Florești, 400524 Cluj-Napoca, Romania; mihail.chira@incerc-cluj.ro; 3 Faculty of Civil Engineering, Technical University of Cluj-Napoca, 15, Constantin Daicoviciu Str., 400020 Cluj-Napoca, Romania; cristina.campian@dst.utcluj.ro

**Keywords:** cementitious composites, TiO_2_ nanoparticles, steel reinforcement, corrosion resistance, open circuit potential, linearization, electrochemical impedance spectroscopy

## Abstract

The aim of this paper was to examine the effects of adding TiO_2_ nanoparticles to cementitious compositions and partially substituting natural aggregates with recycled aggregates consisting of glass, brick, slag, or textolite, and to examine the material’s ability to resist corrosion under the action of chloride ions existent in the environment that attack the steel reinforcement. The results show that the changes in the cementitious composite when it comes to the composition and microstructure influence the formation of the oxide passivating layer of the reinforcement. The addition of TiO_2_ nanoparticles and recycled aggregates impacts the kinetics and corrosion mechanism of the reinforcement. An addition of 3% TiO_2_ was found to be optimal for reinforcement protection. Electrochemical impedance spectroscopy confirmed the results obtained by open-circuit potential and linear polarization tests. The classification of favorable conditions indicates that compositions with recycled aggregates and 3% TiO_2_ are the most effective, with compositions in which the natural aggregates were partially substituted with slag being the most effective.

## 1. Introduction

The demand for built spaces for various industrial activities, commercial purposes, and living spaces is continually increasing, both now and in the long term. While there are trends towards introducing innovative building materials into everyday use, reinforced concrete remains the primary option in terms of material due to its versatility and durability. Despite several research programs developed worldwide over the past 20 years, many coordinated within large international frameworks (e.g., CEB:1992, International Union of Laboratories and Experts in Construction Materials, Systems and Structures (Rilem):1995, Frederiksen:1996, Comité Euro-International du Béton (CEB):1997, European Cooperation in Science and Technology (COST) 509:1997, American Concrete Institute (ACI) 365:2000, Duracrete:2000, Rilem:2000, COST 521:2003), there is still no universally accepted procedure for designing reinforced concrete structures that ensures maximum corrosion protection for the reinforcement. Nevertheless, electrochemical techniques are widely accepted and used to monitor and evaluate the corrosion resistance and degree of corrosion of reinforcement [1].

Reinforced cementitious composites and concrete are some of the most used composite materials in construction since they combine the compressive strength of the concrete with the resistance to tensile stress of the reinforcement. Even though these composites have a long lifespan, they are still susceptible to physio-mechanical and chemical degradation. This can be caused by the external environment (climatic agents, freeze-thaw cycles, carbonation, chloride, sulphate, acid environment, etc.), extreme events (fires, earthquakes, etc.), or biological agents (some micro-organisms growing and invading the cementitious surface). Either way, the degradation of the cementitious matrix and/or the reinforcement can result in a complete loss of strength and safety performance while in use [2]. Generally, for a good cementitious composite layer, the reinforcement is shielded by its specific alkalinity. This enhances the stability of a 5 to 13 nm passivating film primarily containing Fe^3+^ oxides, with Fe^2+^ oxides predominating in the outer layer [2,3]. This passivation layer can be destroyed, and its passivation conditions disturbed. This can be achieved either by the carbonation of the cementitious composite, thus reducing its alkalinity, or by the penetration of Cl^−^ ions to the surface of the metal reinforcement through the porosity and microcracks of the cementitious layer. This generates the corrosion of iron. Therefore, corrosion products with a high specific volume (even 10 times higher than that of iron, the main constituent of steel) are formed, which induces internal stresses in the adjacent cementitious matrix, leading to the occurrence of microcracking right at the junction between the reinforcement and the surrounding cementitious matrix. After these microcracks appear, the corrosive agents penetrate the cementitious composite layer more easily, reaching the metal surface of the reinforcement quicker and in larger quantities. This intensifies the corrosion process until the adhesion between the reinforcement and the matrix is destroyed [2,4,5,6].

Studies regarding the corrosion of reinforcement in cementitious composites show that the most common methods of testing are by electrochemical means (open circuit potential, surface potential measurement, Tafel potentiodynamic polarization measurement, linear polarization resistance measurement, electrochemical impedance spectroscopy). These methods allow both quantitative and qualitative analysis of the phenomenon [2,7], and some of these methods are standardized [8].

Currently, methods of additional corrosion protection, either through the modification of the cement matrix, the use of corrosion inhibitors, the surface treatment of the reinforced electrochemical extraction of chloride ions, electrochemical re-alkalinization, or cathodic protection are studied [9,10,11,12]. Each of these methods has their advantages and disadvantages. More and more research is being carried out to improve them or to identify new methods, especially when it comes to the protection of the reinforcement. Therefore, in recent years, the increasing accessibility of nanomaterials has led to a new trend, exploiting their effect on cementitious composites to improve the corrosion resistance of steel–cement–matrix composites.

The existing literature shows and documents the influence of TiO_2_ nanoparticles on cementitious composites. Among the induced effects, microstructural changes in the hydration products of cement with an up to 50% acceleration of calcium silicate hydrates (C-S-H) and calcium hydroxide (CH) are shown. At the same time, a 15% reduction in tri and bi silicate compounds (C_2_S/C_3_S) has been discovered when compared to the classical case of cement hydration [13,14,15]. These qualitative and quantitative changes in the formation of cement hydration products lead to increased compactness, the densification of the matrix, and changes in porosity with the formation of predominantly closed small-sized pores [16,17,18,19,20,21]. As a result of these changes, there is a reduction of more than 43% in the permeability to gases, water vapors [22], water, or other substances. At the same time, the physico-mechanical performances in terms of strength are simultaneously increased. There have been reports of improvements in mechanical strength when adding 1.5–10% TiO_2_ nanoparticles in cementitious composites, such as an over 45% increase in compressive strength [23]; a 50% [21] to 87% increase in flexural strength [24]; and an over 43% increase in tensile strength [25]. The optimal concentration of TiO_2_ added to the cementitious composites varies from 1% to 10%, the most common percentages being between 3% and 5% [14,21,26,27].

There is a research gap in the existing literature when it comes to the corrosion behavior of these composites. However, according to Rahim and Nair, adding TiO_2_ nanoparticles to the cementitious composite matrix can lower the water absorption coefficient by 59.11% while lowering the corrosion rate caused by sulphate and chloride ions by as much as 75.03% and 49.81% [23]. At the same time, Mohseni et. al. [28] show that an optimal percentage of 1% TiO_2_ nanoparticles in the cementitious matrix reduces chloride diffusion and increases resistance to sulphuric acid attack. Mostafa et al. [7] reported corrosion rate reductions from 545 mm/yr. for the control sample to 483–329 mm/yr. for composites containing 0.5–2.5% TiO_2_ nanoparticles after 2 months of immersion in water, with a corrosion inhibition yield of 11.38% to 39.63%. When testing was conducted for a longer time frame (6 months), the corrosion inhibition was higher than 70%, reaching 72.91% for the 0.5% TiO_2_ nanoparticle sample and increasing with an increasing nanomaterial concentration, with this rising up to 85.09% for the 2.5% TiO_2_ nanoparticles sample. In accordance with these findings, other research groups [19,29,30,31] have also shown that adding a concentration of 1% to 5% wt TiO_2_ nanoparticles in the cementitious composite can reduce chloride ion permeability by a range of 31% to 59%. For concentrations of 1% wt of nanoparticles added to the cementitious composites, there is a significant increase in the carbonation resistance, the depth of carbonation being reduced by 70% compared to the control sample.

Considering all these results, a first research direction could be identified: modifying the cementitious composites at the microstructural level by adding TiO_2_ to improve the composites’ ability to protect the embedded reinforcement. Although an interesting approach, this research direction comes with some challenges: the presence of TiO_2_ nanoparticles in cementitious composites is known to reduce workability; there are increased water requirements for good workability; there are changes in the setting time, the heat for the hydration of cement, and worst case scenario there could be agglomeration of nanoparticles in so-called islands that not only prevent the full exploitation of their potential benefits, but even create weaknesses in the cement matrix, both in terms of mechanical strength and adhesion to the reinforcement [20,21,26,29,32,33]. The most challenging parameter to determine is the optimal ratio of TiO_2_ nanoparticles and how to incorporate them into the cementitious matrix, a process influenced by the specific characteristics of the raw material and the type and characteristics of the nanoparticles.

Apart from research focusing on increasing the sustainability of reinforced cementitious composites, a second research direction can be identified: reducing the environmental impact, by identifying new possibilities for recycling certain industrial wastes and by-products and using them as raw materials in cementitious composites. The presented recycling methods can have several benefits for the performance of the cementitious composite in which they are included, in addition to the benefits of lessening the impact on the environment [34,35]. Ghamari et al. [36] reports that concrete containing glass powders has increased resistance to extreme temperatures and fire. Tang et al. [37] reports a similar benefit when using shredded rubber waste in cementitious composites. Similarly, research suggests that using nanoparticles could lead to the creation of environmentally friendly cementitious composites with excellent durability performance [38,39,40,41]. The scientific literature documents this area extensively, showing encouraging results for the use of recycled aggregates such as waste glass, ceramic bricks, or other materials from building demolition, slag, slurries, plastics, etc. The nature of the recycled waste is one of the most important parameters; therefore, each type of aggregate must be assessed on its own and each presents a certain set of challenges.

Research shows that recycled glass aggregates contribute to changes in the workability, porosity of the cementitious composite, water absorption and mechanical strength, or may or may not have pozzolanic activity. These changes are all dependent on the granularity, composition, surface condition, etc., of the glass [42,43,44,45]. When adding recycled ceramic brick aggregates to the cementitious matrix, an increase in the water/cement ratio (to preserve the workability of the cementitious composite) and an increase in water absorption is observed, along with a reduction in the density and mechanical strength and an increase in water absorption [46,47,48,49,50,51,52,53]. Slag waste, which is defined by its varied and complex oxidative composition, can also influence, both favorably and unfavorably, the micro and macrostructure of cementitious composites and their characteristics [54,55]. The literature is not as extensive when it comes to waste plastics and waste from electrical and electronic equipment (WEEE), but problems with adhesion to the cementitious binder, increased porosity, and decreased mechanical strength are mentioned [56,57,58].

Based on the literature study presented in this work, it can be concluded that the durability of reinforced cementitious composites, generally described as corrosion resistance, is strongly influenced by the specific characteristics of the cementitious matrix (porosity, permeability, water absorption, etc.) and by the addition of TiO_2_ nanoparticles, which allow a better/weaker penetration of the corrosive agent at the surface of the reinforcement. Regarding the durability of the construction and the influence on the environment, each of these variables has benefits and drawbacks. The literature is currently rich in results from multiple studies addressing the impact of using TiO_2_ nanoparticles and recycled waste aggregates on the physico-mechanical performance of cementitious composites; however, it is lacking an electrochemical analysis of these raw materials’ effects on metallic reinforcement corrosion. Therefore, the aim of this work was to analyze the effects of adding TiO_2_ nanoparticles and partially substituting natural aggregates with recycled waste or industrial by-product aggregates on the corrosion resistance of steel reinforcement embedded in the cementitious matrix. The goal of this interdisciplinary approach is to complete the body of knowledge in the field and help create a favorable environment for the development of new sustainable approaches in the construction industry. This is especially important since, according to the National Association of Corrosion Engineers (NACE), the cost of corrosion is estimated to be USD 2.5 trillion annually, or 3.4% of the world’s gross domestic product; these are costs that can be reduced by 15% to 35% by choosing appropriate methods of protection and increasing corrosion resistance [59].

## 2. Materials and Methods

The impact of TiO_2_ nanoparticles on the durability of reinforced cementitious composites is measured as the strength of the steel reinforcement under chloride ion attack. Cylindrical specimens of a cementitious composite in which a steel specimen was inserted for concrete reinforcement were tested by electrochemical methods. The steel that is often utilized for such reinforcement, therefore used for testing in this work, is the 8 mm diameter B500C periodic profile steel reinforcement. This steel has the following characteristics: yield strength 500 N/mm^2^, tensile strength 550 N/mm^2^_,_ and elongation at break (A_gt_) min. 16%.

**Figure 1:**
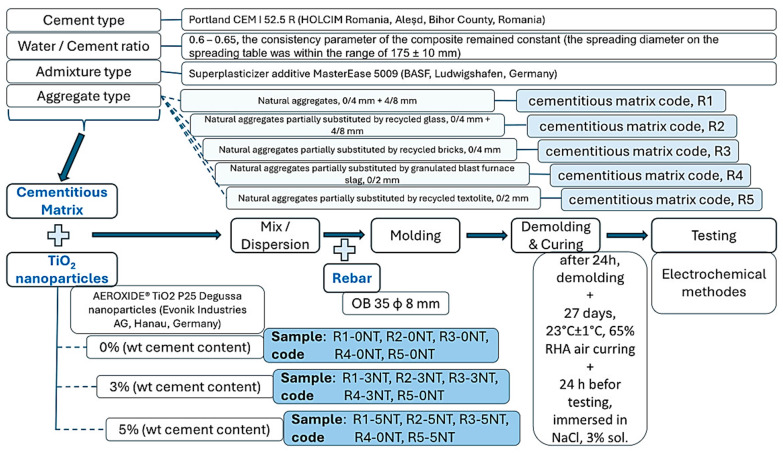
Methodology.

The materials used for the production of the cementitious composites were Portland cement CEM I 52.5 R (HOLCIM Romania, Aleșd, Bihor County, Romania), characterized by a Portland clinker content of min. 95% and a compressive strength of min. 52.5 N/mm^2^; tap water; a mixture of natural and recycled waste or industrial by-product aggregates of local origin; water-reducing superplasticizer MasterEase 5009 (BASF, Ludwigshafen, Germany); and TiO_2_-type AEROXIDE^®^ TiO_2_ P25 Degussa nanoparticles (Evonik Industries AG, Hanau, Germany) characterized by an average grain size of 25 nm, a specific surface area of 35–65 m^2^/g and a purity of 99.5%, containing mostly the tetragonal anatase form of TiO_2_ crystallization. The mix design methodology and the characteristics of the materials are presented in Figure 1.

### 2.1. Aggregates Characterization and Importance

**Figure 2:**
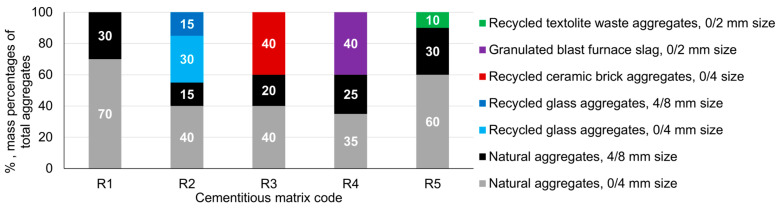
Aggregate ratios (mass % of total aggregates) in the composition mixture of the cementitious composite.

**Figure 3:**
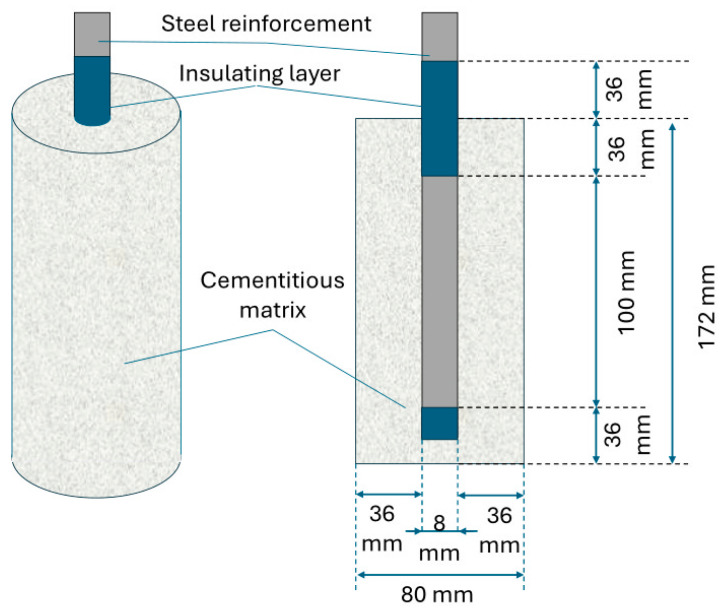
Cement composite specimen with embedded reinforcement—front view (**left**) and section (**right**).

The type of aggregate used in the matrix is of great importance. To determine the impact on the corrosion resistance of the reinforcement embedded in the cement matrix natural aggregates (NAs), the 0/4 mm and 4/8 mm granular classes were used initially. The mixing ratios (Figure 2) were determined based on previously published preliminary research [60,61]. Subsequently, these natural aggregates were partially substituted with aggregates derived from recycled waste or industrial by-products, the optimal substitution ratio also being established based on the same preliminary studies. The characteristics of the recycled aggregates, their nature, and the method used to determine each parameter are shown in Table 1. Recycled glass waste aggregates (RGAs) with a 0/4 mm and 4/8 mm grain size class, recycled ceramic brick waste aggregates (RBAs) with a 0/4 mm grain size class, granulated slag aggregates (GBAs) with a 0/2 mm grain size class, respectively and recycled textolite waste aggregates from electronic equipment (RTAs) with a 0/2 mm grain size class were the substituents used. Their ration within the cement matrix is presented in Figure 3.

Firstly, a control composition (R1-0NT), without TiO_2_ particles (NT), was produced with the following characteristics: cement content 366 kg/m^3^, total amount of natural aggregate mix 1577 kg/m^3^, water/cement ratio 0.6, and amount of superplasticizer additive 0.5% of cement amount. Consequently, mixtures in which natural aggregates were partially substituted with recycled aggregates (R2-0NT, R3-0NT, R4-0NT, and R5-0NT) were prepared. To analyze the impact of NT on the corrosion behavior of the reinforcement embedded in the cementitious matrix, for each previously prepared mixture, corresponding compositions were produced by adding 3%NT (R1-3NT, R2-3NT, R3-3NT, R4-3NT, and R5-3NT) and 5%NT (R1-5NT, R2-5NT, R3-5NT, R4-5NT and R5-5NT). The mechanical testing of these mixtures had been previously performed and the results had been published by the authors in several other papers [60,61,64].

The available literature mentions the importance of the aggregate’s nature and that their use in the production of cementitious composites may result in changes in the workability. This can be assessed as a risk when working with such materials. [17,18,65,66,67,68]. To the same extent, the addition of TiO_2_ nanoparticles increases the water required to maintain the workability of the mixtures. To reduce these risks, the additional condition of constant workability was applied to all mixtures. This condition is measured by a constant 175 ± 10 mm diameter on the spreading table (according to EN 1015-2) [69]. Thus, by adding water when the conditions required it, the water/cement ratio increased from 0.6 (RT-0NT) to 0.65 (maximum value recorded) for the sample prepared with 5% NT and recycled textolite aggregates (RT-5NT).

Another risk mentioned by previous research [33,70,71] is that of the inhomogeneous dispersion of nano-TiO_2_ particles. This results in the formation of islands comprising agglomerated NT particles in the cementitious matrix. It is well known that NT particles tend to agglomerate because of their large specific surface area and unsaturated binding sites [72,73,74]. Thus, there is a risk of composite inhomogeneities. To obtain a homogenous composite matrix, there are several methods of prevention that have been reported in previous works [75]: dry mixing (per-homogenization), dispersion in suspension, or the use of surfactants have given good results in terms of homogeneity [14,76]. More complex methods such as combining the dispersion in suspension with an ultrasound bath have also given good results [77]. The pre-mixing of the NT with the cement at a low speed with only ½ of the water quantity was chosen in order to prevent island formation, a method with good results and mentioned in previously published work [60,78]. After the initial mix, the superplasticizer, the water and aggregates were added to the mixture. The efficiency of this working procedure in terms of the homogeneous dispersion of NT in the composite matrix was previously verified and demonstrated using SEM-EDX analysis [60,78].

### 2.2. Cementitious Composites Characterization and Sample Preparation

The characterization of the prepared cementitious composites was carried out using standardized methods and is shown in Table 2. Water absorption was considered as an indirect indicator for studying the way in which the saline solution, containing corrosive chloride ions, penetrates the cementitious composite layer and succeeds in initiating corrosion at the surface of the metal reinforcement. The KERN FKB 36K0.1 balance (KERN & SOHN GmbH, Balingen, Germany) with an accuracy of 0.1 g and ELE paddle mixer (ELE International Ltd., Milton Keynes, UK) were used for dosing the raw materials and preparing the cementitious compositions. All raw materials were conditioned prior to use for 24 h in the laboratory at 23 ± 1 °C.

Each composition was poured into molds with an inner diameter of 80 mm, in which a BC500 metal reinforcement of ϕ8 mm in diameter (OAM Ózdi Acélmüvek Kft., Ózd, Hungary) was inserted vertically and centrally so that at any point the thickness of the cementitious composite coasting was constant. Figure 3 shows the cement composite specimen with the embedded reinforcement used both in full view and cross-section, as well as the dimensional characteristics of the sample.

The length of the metal specimen was set so that the part that was not embedded in the cementitious composite allowed connection to the test equipment. The metal reinforcement specimen was prepared prior to use by cleaning and removing any pre-existing rust, sealing the lower end and the upper area crossing the cementitious surface with an epoxy resin. These sealing operations were carried out to delimit an equal and controlled area of exposed metal surface (5024 mm^2^) and to avoid differential aeration corrosion in the area where the reinforcement protrudes from the cementitious material, which would otherwise have been in contact with the saline solution used as the electrolyte in the electrochemical cell. It is well known that cementitious matrices exhibit a relatively uniform distribution of both closed and open porosity. This porosity plays a crucial role in the study of corrosion, the phenomena affecting the reinforcement embedded in cementitious composites, as it facilitates the transfer of corrosive agents and oxygen to the reinforcement surface. Consequently, the experimental procedure was designed based on the Gaussian distribution principle. Five identical specimens were produced and tested, with the outliers being discarded. From the remaining specimens, the one with median results was chosen, representing a sample that was neither in the most favorable nor the most unfavorable range.

The specimens were de-molded and conditioned in laboratory conditions ((23 ± 1) °C and 65% RH). At 28 days of age, they were submerged in a 3% NaCl saline solution for 24 h to saturate the cementitious matrix with electrolyte and tested for corrosion resistance.

### 2.3. Electrochemical Analysis and Experimental Set-Up

**Figure 4:**
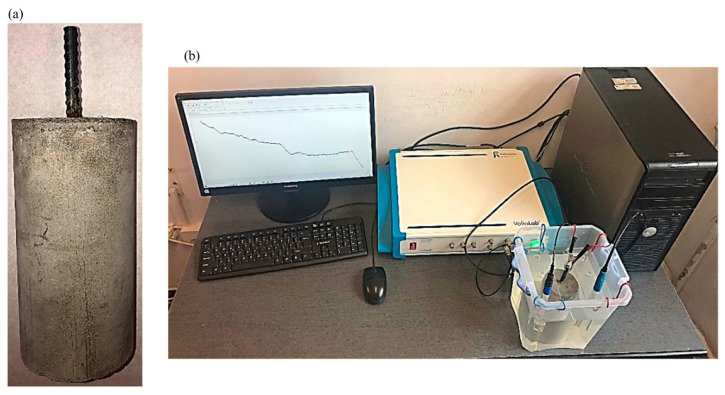
(**a**). Cement composite specimen with embedded reinforcement. (**b**). Experimental stand with the electrochemical cell for testing.

Electrochemical techniques were used to characterize the behavior of the embedded reinforcement in each of the 15 different types of cementitious compositions (Figure 4). A 3% NaCl aqueous saline solution represented the electrolyte used for testing, and the working electrode was represented by the analyzed reinforcement, with a Ag/AgCl (3 M KCl) reference electrode (Radiometer Analytical) and a Pt counter electrode (Radiometer Analytical). The specimen was immersed in the electrolyte so that the upper surface of the cement cylinder was covered with a sufficient layer of electrolyte, but the electrolyte did not exceed the epoxy-resin-insulated area of the reinforcement (it did not come into direct contact with the exposed surface of the reinforcement in order to not initiate the phenomenon of corrosion by differential aeration). The electrodes were connected to a VoltaLab PGZ 100 potentiostat (Analytical Radiometer, Copenhagen, Denmark). The interpretation and analysis of the results were performed with Volta Master 4 Electrochemical Software and ZView Software (version 3.4).

Three types of electrochemical tests were conducted:

➢ **Open circuit potential (OCP)** tests were carried out for 1200 min, which made it possible to identify the passive oxide layer’s creation and destruction tendencies and conduct a qualitative investigation of the phenomenon.
➢ **The linear polarization** test offers quantitative information on the corrosion process and its kinetics. For this study, the potential was swept at a rate of 10 mV/min, over the range ±500 mV from the open circuit potential value. Based on the experimentally obtained diagrams, in Tafel interpretation, the main kinetic indicators were recorded: corrosion potential, corrosion current, corrosion rate, anodic and cathodic slope, and polarization resistance.
➢ **Electrochemical impedance spectroscopy (EIS)** measurements were performed to evaluate the mechanism of the corrosion process. The alternating current frequency range used for the studies started at 100 kHz and went up to 100 mHz, with an amplitude of 10 mV. Plots in the Bode (logarithmic representations for impedance-frequency modulus and phase angle–frequency) and Nyquist (an imaginary component of impedance vs real component) representations were created using the impedance spectra that were recorded at the open-circuit potential.

After the electrochemical testing, the specimens were split in half along their length and the surface of the reinforcement was analyzed by optical microscopy using the LEICA SAPO optical microscope (Leica Microsystems, GmbH, Wetzlar, Germany), as opposed to using an SEM analysis. There is no denying that the latter would give detailed information about the sample surface, kinetic energy, and dissipation. However, this work reports on results that are part of a larger research project that is still ongoing. SEM analysis will be conducted at later stages of the project when more samples will be produced and tested. All tests were carried out under laboratory conditions at (23 ± 1) °C and 65% RH.

## 3. Results and Discussion

### 3.1. Influence of the Cementitious Matrix on the Thermodynamics of the Reinforcement Corrosion Process Analyzed by the Open Circuit Potential Recording Method

The results of the open-circuit polarization (OCP) give qualitative information on the corrosion process occurring at the surface of the reinforcement. This method is based on measuring the natural electrochemical potential of a metal electrode in a given environment without applying any external current. The OCP represents the equilibrium potential where the anodic and cathodic reactions occurring on the metal surface are balanced.

**Figure 5:**
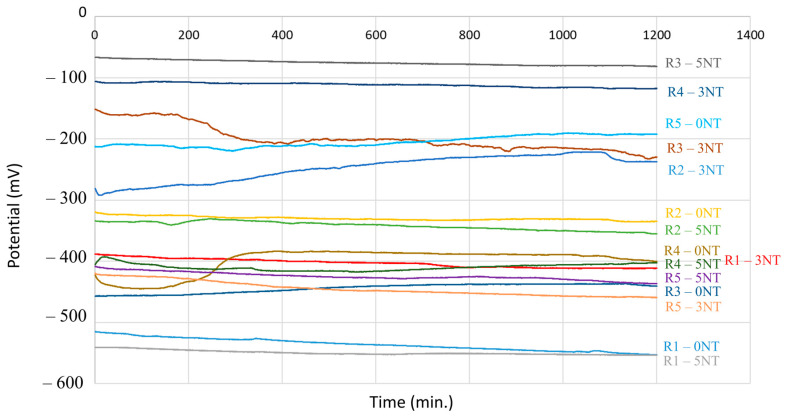
Open-circuit potential evolution.

Figure 5 shows that a voltage drops from −515 mV to −553 mV was observed for the R1-0NT sample. This corresponds to the oxidation of the steel reinforcement and the diffusion of iron ions into the solutions. At minutes 344 and 1071, there is a tendency for positivity, which would indicate the formation of the oxide layer, which rapidly dissolves due to its unstableness. Looking at sample R1-3NT, there is a negative potential from −388 mV to −410 mV in the first 800 min after the beginning of the measurement, as explained by the corrosion of the reinforcement and the diffusion of iron ions into the solution. From the 800 to 870 min mark, there is a slightly positive shift in potential from −410 mV to −408 mV, indicating the formation of the oxide layer; however, the slight negative trend that follows shows its destruction. From minute 970 onwards, the recorded potential has a relatively constant value, indicating the stability of the iron oxide passive layer.

When analyzing sample R1-5NT, the closed-circuit potential shows a negative shift from −541 mV to −552 mV over a period of 634 min. During this time, corrosion of the steel layer occurs and, from this point onwards, there is a constant tendency for the potential to increase to positive values, showing stability in the passivation layer, although the graph shows small fluctuations. These fluctuations translate to the slight destruction of the oxide passivating layer, followed by the formation of an additional layer. Taking all the information provided by the OCP measurement into account, it can be concluded that in the case of cementitious mixtures prepared with NA, sample R1-3NT shows the best stability from a thermodynamic point of view; it has the lowest potential decrease and the general tendency for stabilization.

Comparing samples R1-0NT, R1-3NT and R1-5NT, the results show that the inclusion of NT in the cement matrix improves the reinforcement’s corrosion resistance. Although it is not possible to conclude that a higher concentration of NT will improve the thermodynamic stability of the metal surface, it is possible to estimate an optimal range for the NT addition to determine the maximum degree of protection offered by the matrix to the thermodynamic stability of the reinforcement.

Samples in which NA has been partially substituted with RGA without the addition of NT (R2-0NT) have an OCP value showing continuous negativity until the 757 min mark, corresponding to the oxidation of steel and the release of iron ions into the solution. Between the 757 and 1098 min mark, a potential positivity is observed, representing the formation of the oxide layer followed by its sudden dissolution. Sample R2-3NT has a potential that continuously shifts towards positive values up to 1139 min, after which a slight shift towards negative values occurs that lasts until the value is stabilized. This evolution shows the formation of the oxide layer, as well as its partial dissolution. This sample shows better stability from the thermodynamic point of view when compared to sample R2-5NT, which presents a continuous trend towards negative potential values that indicate the oxide layer formed is not stable. The results show that the addition of NT can contribute to the improvement of the corrosion resistance behavior of the reinforcement, with an optimal range of NT content for the highest protection.

In the case of partially substituting NA with GBA within the matrix, some aspects are potentially observed due to the importance of the substituent’s nature, as previously mentioned. When analyzing the reinforcement that contained no NT (R4-0NT), the OCP has a negative trend between the 0 and 102 min interval, showing an obvious tendency for corrosion. The potential shifts towards positive values (form −444 mV to −383 mV) in the time interval from 102 to 385 min indicate a tendency to stabilize and the formation of the protective oxide layer. After that, the potential is relatively constant between the 358 and 1002 min mark, with a slight negative shift towards the end of the measurement from −389 mV to −400 mV. As a result, it is evident that the steel surface corrodes in the first phase, releasing iron ions into the solution; this is followed by the formation of a stable oxide layer that eventually dissolves partially at the conclusion of the test time.

When looking at samples with 3% NT (R4-3NT), there is a successive shift of potential from negative to positive values and vice versa, the general trend being towards negative values. This indicates the successive formation and dissolution of the oxide layer, with the passivation layer being unstable. On the other hand, in the case of the R4-5NT sample, there is a potential positivity (formation of the oxide layer) followed by a sharp negativity from −392 mV to −417 mV between the 19 and 557 min mark. The next time interval (557–1200) shows a continuous potential increase from −417 mV to −402 mV; therefore, it can be said that the second formation of the oxide layer is more stable than the first one. For the samples that contain GBA and NT, it can be said that R5-5NT has the best thermodynamic stability, considering the position of the trendlines within the graph (Figure 5). It is necessary to identify the optimal NT range in the case of these cementitious composites so that the benefits are significant and obvious in terms of the thermodynamic stability of the reinforcement. The clear proximity of the recorded R4-5NT sample and the obvious shift towards positive values of the R4-0NT sample attests to this fact.

For samples produced with RTA as a substituent, there is a continuous shift towards negative values in the OCP, indicating the dissolution of the oxide layer and metal instability. The potential of the R5-0NT sample is successively negative and positive, but the general trend is still positive, indicating the relative stability of the oxide passivating layer. Furthermore, the placement of the characteristic curve for R5-0NT in a region much shifted towards the positive range, compared to the plots of the R5-3NT and R5-5NT samples, in conjunction with the specific characteristics of these composites can be considered a counterargument in terms of the hypothesis suggesting the improved corrosion protection of reinforcement embedded in such cement matrices. Table 2 shows that the highest coefficient for water absorption is characteristic of these samples and indicates the ease with which chloride ions are transported within the saline solution, reaching the reinforcement’s surface in a larger quantity compared with the other tested composites.

All these conclusions support a hypothesis that indicates that there are several factors that influence the stability of the passivation layer, from a thermodynamic point of view. Therefore, the raw material (a variation in the nature of the aggregates) influences the physical characteristics of the cementitious matrix, a fact supported by previous research [60,78]; it also influences the degree of protection that this matrix provides the reinforcement against the chloride ions by hindering or facilitating their penetration and overflow at the interface between the matrix and the metal surface. Simultaneously, adding NT causes modifications to the cementitious composite matrix, serving a similar purpose. However, just as the physico-mechanical characteristics of the cementitious composites follow the Gaussian curve rather than a linear one, there is no linear link between the optimal NT addition and the degree of protection, translating into the possibility of forming and having a stable passive layer of oxide on the surface. This highlights the need to identify the optimal NT content for each raw material situation. For the samples analyzed during this research, it can be estimated that the optimum NT addition is around 3% (wt. vs. the amount of cement), with no justification for a 5% addition in terms of benefit; moreover, it can also induce disadvantages (composite with RGA and GBA substitutes).

### 3.2. Influence of the Cementitious Matrix on the Kinetics of the Reinforcement Corrosion Process Analyzed by the Linear Polarization Method in Tafel Interpretation

The linear polarization method is a corrosion-monitoring technique that allows corrosion rate measurements to be performed in real-time. The principle of this method stands on the inverse proportionality relation between the corrosion current density and the polarization resistance of a metal in an electrolyte. It is a quick non-destructive method for corrosion rate assessment.

**Figure 6:**
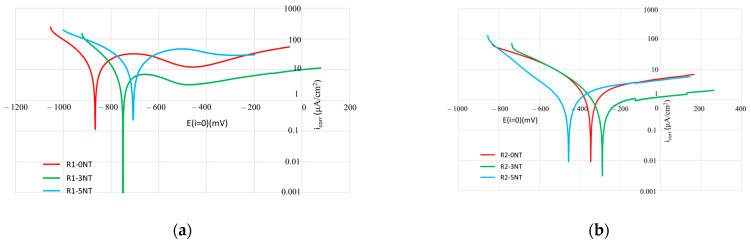

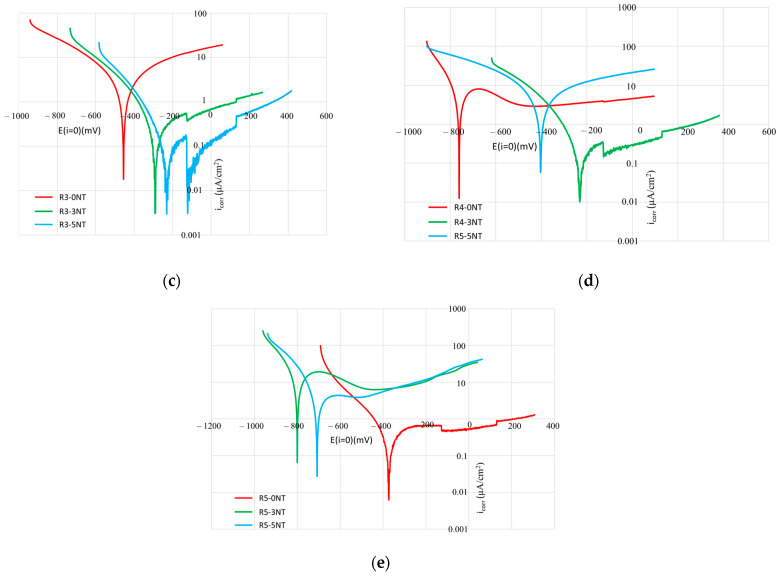
Linear polarization diagrams in Tafel interpretation—(**a**) mixtures with NA; (**b**) mixtures with NA partially substituted with RGA; (**c**) mixtures with NA partially substituted with RBA; (**d**) mixtures with NA partially substituted with GBA; (**e**) mixtures with NA partially substituted with RTA.

The kinetic indicators of the corrosion (E—corrosion current, R_p_—linear polarization resistance, i_corr_—current density, β_a_—anodic slope, β_c_—cathodic slope, v_corr_—corrosion rate) are displayed in Table 3, while Figure 6 shows the linear polarization diagrams in the Tafel interpretation.

Looking at the data gathered for the reinforcement embedded in cementitious mixtures prepared with NA (R1-0NT, R1-3NT, R1-5NT), it is observed that the corrosion current is the lowest when adding the highest amount of NT. At the same time, the anodic slope (β_a_) is lowered in comparison to the values of the same parameter in samples R1-0NT and R1-3NT. These indicate that the steel reinforcement introduced into the cement mixture with the highest amount of NT has a higher activation energy corresponding to the dissolution process. The corrosion potential (E (i = 0)) shifts towards positive values and the corrosion current density (i_corr_) decreases with the increase in NT addition, which points towards a decrease in the kinetics of the process. This can be correlated with the corrosion rate parameter (v_corr_), which is decreasing, reaching half of the rate of the control sample R1-0NT for the composition with 3% NT, and which is 17 times lower for the composition with 5% NT.

For reinforcements embedded in a cementitious composite in which NA is partially substituted with RGA (R2-0NT, R2-3NT, R2-5NT), the same observation can be made; the sample with the maximum addition of NT has the corrosion current and the anodic slope with the lowest values. This indicates again that for the sample containing the highest amount of NT, the activation energy of the reinforcement, corresponding to the dissolution, is higher than the one of other samples. In corroboration with the open-circuit potential (OCP) measurements, it can be assumed that the reaction rate in this sample is so high that the electroactive species cannot reach or be removed from the surface of the steel reinforcement at a sufficiently high rate. The reaction rate becomes controlled by the diffusion process, resulting in a small corrosion current. On top of that, the high values of the polarization resistance suggest the existence of a large agglomeration of iron ions around the reinforcement. For the R2-3NT sample, there is a shift towards positive values for the corrosion potential and a decrease in the corrosion current, unlike in the case of sample R2-0NT. This phenomenon signifies a reduction in the process kinetics, while for R2-5NT, the Tafel curve shifts in the opposite direction.

The position of the Tafel plots for the R2-0NT and R2-5NT samples (Figure 6b) indicates an increase in the probability of corrosion rather than an acceleration of processes, quantified by the corrosion rate value; the corrosion rate potential of the R2-5NT sample is shifted towards negative values. This is once again evidence that an excess of NT is not beneficial and that there is a need to identify the optimum NT addition in each case. Taking all this information into account, as well as the OCP result, it can be concluded that sample R2-3NT has a better corrosion resistance, meaning that this sample’s composite matrix gives the best corrosion protection to the embedded reinforcement.

For samples made with cementitious composites in which NA is partially substituted with RBA (R3-0NT, R3-3NT, R3-5NT), the anodic slope of the R3-0NT sample is lower than the other samples, indicating that, for the R3-0NT sample, the steel reinforcement has a higher activation energy corresponding to dissolution than the other samples. Although the corrosion current of samples R3-3NT and R3-5NT is lower than that of sample R3-0NT, their anodic slopes are higher, and their polarization resistances are significantly higher. This can be explained by the existence of a large agglomeration of ions around the armature shielding it, resulting in a small corrosion current; this is small not because the oxide layer formed is stable, but because of the shielding of the reinforcement by iron ions released in solution. Based on the positioning of the Tafel diagrams relative to the NT-free sample (R3-0NT, Figure 6c), however, it can be said that the addition of NT to the composite matrix favors better reinforcement behavior as a response to chloride ion attacks.

Cementitious mixtures in which NA is substituted with GBA show similar results to previously discussed samples; the anodic slope of the sample with the maximum addition of NT (R4-5NT) is lower than the other samples, indicating the higher activation energy of the dissolution. Sample R4-3NT shows a low corrosion current, assumed to be due to the high ion density released into the solution shielding the reinforcement and not due to the formation of a stable protective oxide layer. The positioning of the Tafel diagrams (Figure 6d) in areas of obviously positive potentials, and the lower corrosion current for the R4-3NT sample but the higher one for the R4-5NT sample, also give multiple insights into the phenomenon. Therefore, it can be said that the probability of corrosion decreases with the addition of NT, which is supported by the shift in the corrosion potential towards positive values; however, the kinetics of the phenomenon is reduced only in the case of the R4-3NT sample, for which the corrosion current is lower.

In the case of reinforcements embedded in cementitious composite matrices in which NA was partially substituted with RTA (R5-0NT, R5-3NT, R5-5NT), the results are, as in the OCP tests, contradictory. The activation energy of the dissolution is higher for the R5-0NT sample, with the anodic slope being lower than in other samples. The polarization resistance for this sample is higher, corroborating with the OCP results that these high values are due to the shielding of the reinforcement by the iron ions and the formation of an oxide layer. Considering the physical characteristics of the cementitious matrix and the result of the linear polarization, the initial hypothesis that the cementitious composite layer offers good corrosion protection to the reinforcement is not proven to be accurate for the RTA substituent. This is probably due to the high porosity of RTA, which allows a significant amount of chloride ions to penetrate the metal surface.

Based on all the information provided, from the perspective of the process’ kinetics, it is safe to say that the kinetics are also influenced by the composition of the cementitious matrix, the existence of the partial substitution of NA with recycled waste aggregates, and the addition of NT. The results indicate that an addition of 3% NT provides greater benefits than using a higher amount (5% NT). This can be explained by factoring in the effects of the added NT on the matrix density, porosity and permeability.

### 3.3. Influence of Cementitious Matrix on the Mechanism of the Reinforcement Corrosion Process Analyzed by Electrochemical Impedance Spectroscopy Method

Impedance spectroscopy is a quantitative method that records the response of the metal surface to the induced excitation. It offers information about the kinetics and the mechanisms of the tested electrochemical system, which are widely used in corrosion studies. During an EIS test, an AC current is applied at different frequencies to a system in equilibrium. The response of the system to this perturbation is recorded, and with the obtained data, an equivalent circuit can be simulated to better describe the processes taking place within the system.

The working potential was chosen to be the open-circuit potential value due to the thickness of the oxide layer not being affected at this value. By analyzing the Nyquist and Bode plots, qualitative information about the layers of corrosion products that form on the surface of the armature (working electrode) was obtained, and by mathematically modeling the corrosion process using an equivalent circuit, quantitative information was obtained. Thus, the ohmic resistance of the electrolyte medium, the polarization resistance at the electrode/solution interface, and the capacitance of the double layer at the interface were determined. On top of that, the polarization resistance is an indicator used in evaluating the corrosion mechanism. This parameter is related to the corrosion current by the inverse proportional relation.

**Figure 7:**
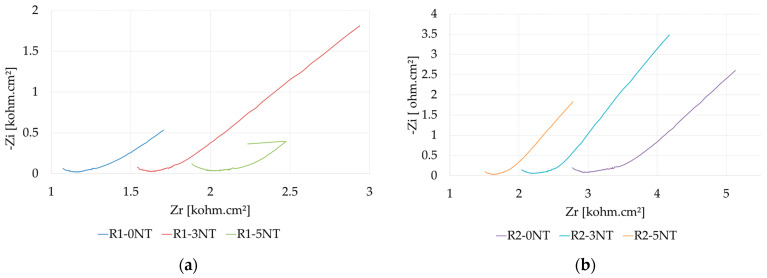

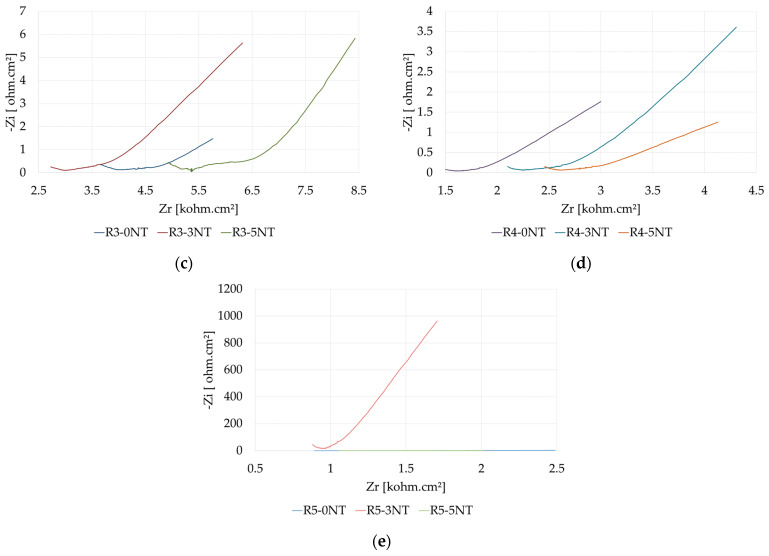
EIS diagrams in Nyquist interpretation—(**a**) mixtures with NA; (**b**) mixtures with NA partially substituted with RGA; (**c**) mixtures with NA partially substituted with RBA; (**d**) mixtures with NA partially substituted with GBA; (**e**) mixtures with NA partially substituted with RTA.

**Figure 8:**
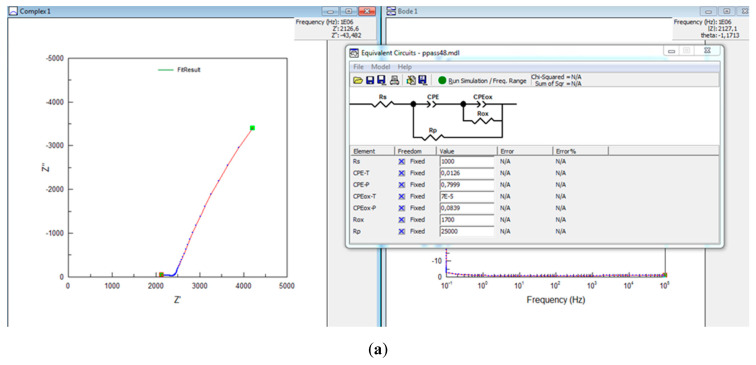

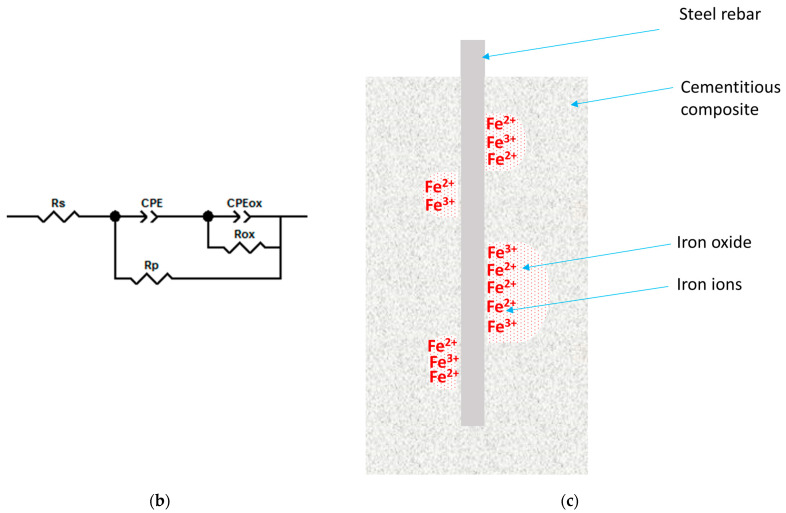
(**a**) Example of how to analyze the experimental results in Nyquist representation (screenshot); (**b**) Mathematical modeling of the corrosion process using the equivalent electrical circuit, characteristic of all the samples tested experimentally; (**c**) Hematic representation of iron ion accumulation at the steel reinforcement–composite matrix interface.

The experimental results are presented in Figure 7 and Figure 8 and Table 4. These show the plots mainly as a straight line, indicating that the reaction kinetics are large and characterized by mass transfer. The absence of the semicircle area indicates the absence of the slow stage within the reaction kinetics when the protective oxide layer is usually formed.

The identification of the electric circuit equivalent to the best phonon is presented in Figure 8b, where the CPE is a constant phase element in which the CPE-T has the dimensions of a capacitor that has “reinforcement” formed by iron ions that are practically on the surface of the steel armature and electrons left by the ions in the metal. The electric charge is characterized by the CPE-P factor; the closer its value gets to unity, the more localized the charge. Other parameters determined are CPE_ox_, corresponding to the iron oxide layer formed; R_ox_, representing the resistance of the oxide layer; R_p_, representing the polarization resistance of the armature; and Rs, representing the resistance of the solution. The values for these parameters are detailed in Table 4.

Analyzing the data in its entirety, it can be said that an accumulation of iron ions occurs on the surface of the reinforcement, which is surrounded by a porous iron layer, the electric charge being unlocalized (Figure 8c). The large variations in the polarization resistance for the studied samples are due to both the iron ions on the surface and the oxide layer, but also due to the chemical composition of the samples and the pore size.

When examining the experimental results and considering the nature of the aggregates, it can be said that the roughly equal values of the oxide layer resistance (R_ox_) suggest that these oxide layers have roughly equal densities, and as a result, the resistance they provide against the displacement of iron ions is roughly equal. On the other hand, the modification brought about by the cementitious matrix’s aggregate nature does not significantly affect the compactness of the oxide layer formed on the metallic surface of the reinforcement. At the same time, the polarization resistance (R_p_) varies greatly once the NA is partially substituted with recycled waste aggregates. It can be noted that all the polarization resistances for the embedded reinforcement in the composites prepared with the partial substitution of NA and without the addition of NT (R2-0NT, R3-0NT, R4-0NT, R5-0NT) are significantly higher than the characteristic value recorded for the case of the R1-0NT composite. This suggests that altering the cementitious matrix greatly strengthens the matrix’s defense against oxygen and electrolyte intrusion, which cause corrosion. This resistance can be explained by variations in the microstructure of the cement stone, including its adhesion to the metal surface, as well as variations in porosity brought on by variations in the nature of the aggregates.

When the experimental data are analyzed considering the impact of the NT addition on the cementitious mixtures, it becomes clear that, for aggregates of the same type, the highest polarization resistance (R_p_) is observed at 3% NT addition. Therefore, a high polarization resistance that induces a hindered evolution of the corrosion phenomenon is a convenient situation from the durability point of view, and therefore this amount of NT addition is favorable. The exception to this general observation is when NA is partially substituted with RTA, where the addition of NT reduces the polarization resistance irrespective of the number of nanoparticles added. This addition is found to have a positive influence on composites made with NA if analyzing the resistance of the oxide layer (R_ox_). This parameter increases, which indicates the densification of the oxide layer, with the best result being recorded for the 3% NT addition (R1-3NT). Nanoparticle addition shows either an increase or a decrease in this parameter (R_ox_) for the other composites.

All this information combined leads to the conclusion that adding 3% NT to cementitious composites is the most practical choice in terms of the embedded reinforcement’s corrosion behavior and, in turn, the system’s durability.

### 3.4. Metal Surface Analysis

**Figure 9:**
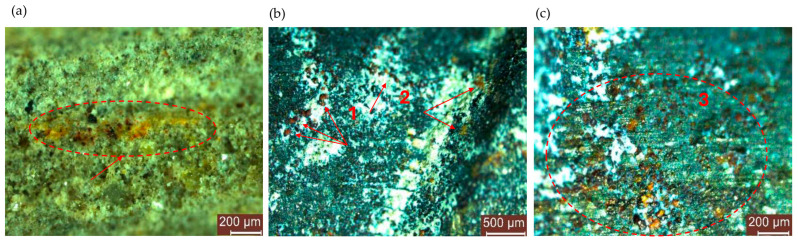
Microscopic analysis of the surface of the reinforcement embedded in composites with NA, after testing. (**a**) Migration of corrosion products into the adjacent cementitious composite matrix (the red circle marks the area of migration of corrosion products); (**b**) Corrosion products identified in the adjacent area (1) or on the reinforcement ribs (2); (**c**) Corrosion products identified in areas with surface defects on the reinforcement (3).

**Figure 10:**
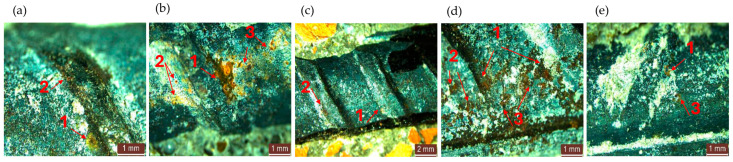
Microscopic surface analysis of embedded reinforcement in composites with NA partially substituted with RGA (**a**,**b**), with RGA (**c**), with GBA (**d**) and with RTA, (**e**) with an example of corrosion products identified in the adjacent area (1) or on the reinforcement ribs (2), or corrosion products identified in areas with surface defects on the reinforcement (3).

After being tested by electrochemical methods, the specimens were split to expose the reinforcement to visual microscopic analysis. Representative images are shown in Figure 9 and Figure 10. In general, areas are formed on the reinforcements’ surface where the iron oxide layer is locally evident, with its migration into the adjacent concrete matrix also being recorded (Figure 9a). Reinforcement corrosion is more frequently localized on the rib area of the reinforcement (Figure 9b) or in the area adjacent to the rib (Figure 9b). Another area that favors the deposition of corrosion products is represented by roughened surface defect areas (Figure 9c), where the metal surface is not smooth and shows concave-type defects. A possible explanation for this phenomenon may be that these concavities exhibit a higher probability of lattice defects and electrolyte deposition, and that accumulation is favored.

## 4. Conclusions

The aim of this work was to examine the effects of adding TiO_2_ nanoparticles to cementitious mixtures and those of partially substituting natural aggregates with recycled waste glass, brick, slag, or textolite on the corrosion resistance of the steel reinforcement embedded in these mixtures when chloride ions are present.

This paper represents a significant contribution to the subject because of its interdisciplinary approach. The kinetics and mechanisms of the corrosion processes that occur on the surface of the metal reinforcement embedded in the cementitious composite are addressed within this study, while accounting for the microstructural modification of the cementitious matrix, which can be caused by the presence of TiO_2_ nanoparticles or by the nature of the aggregates. Therefore, the research results revealed the following:

➢ By analyzing the process from a thermodynamic point of view, there are multiple factors that influence the stability of the passive oxide layer formed on the metal reinforcement surface. First, modifications to the raw material used to produce the cementitious matrix (in this case, a partial substitution of NA with aggregates derived from the recycling of industrial wastes and by-products) will affect the properties of the composite and either increase or decrease the likelihood that a passive oxide layer will form and remain on the surface of the reinforcement. In the same way, the cementitious matrix undergoes structural alterations upon the addition of NT. An additional determining element is the quantity of NT added to the composition; based on the cases examined, it can be approximated that the ideal NT addition is approximately 3% (wt relative to the amount of cement).
➢ By analyzing the kinetics of the corrosion process of reinforcement embedded in cement matrices in the presence of chloride ions, it is possible to conclude that the cement matrix’s composition, the presence of partial NA substitution with recycled waste aggregates, and the addition of NT all have an impact. Therefore, in the scenarios under consideration, the findings suggest that adding 3% NT (weight relative to cement volume) is the optimum option for providing the embedded reinforcement with the best corrosion protection.
➢ The results in terms of electrochemical impedance spectroscopy also support the hypothesis that an addition of 3% NT is preferable, but also the conclusion that the partial substitution of NA induces effects on the corrosion of the embedded reinforcement, this time also in terms of the process mechanism. For all the working variants, the same equivalent electrical circuit was identified, and it can be said that an accumulation of iron ions occurs on the surface of the reinforcement, which is surrounded by a porous iron layer, with the electric charge unlocalized. The large variations between the values of the polarization resistance recorded for the studied samples are due both to the iron ions present on the metal surface and oxide layer and due to the composition, microstructural characteristics, and porosity of the cementitious composite layer covering the reinforcement.
➢ Combining all the aspects of thermodynamics, kinetics, and corrosion mechanism, a classification in terms of the favorable conditions allowing the good strength of the embedded reinforcement in cementitious composites would optimally indicate the mixtures with NA partially substituted with GBA and 3% NT (R4-3NT) and partially substituted with RBA with 3% (R3-3NT) and 5% NT (R3-5NT).

Based on the presented information, future research can be conducted to further advance knowledge in the field. For example, the analysis of the kinetics and mechanism of steel reinforcement corrosion in cementitious composites containing recycled aggregates and/or nanomaterials can be expanded to include new reinforcement types, new approaches to recycling aggregates and nanomaterials, as well as new corrosive environments.

## Figures and Tables

**Table 1:** Characteristics of the used aggregates.

Aggregate Type	Max. Grain Size [mm] EN 933-1 [61]	Density [kg/m^3^] EN 1097-3 [62]	Inter-Granular Porosity (%) EN 1097-3 [62]	Water Absorption Coefficient (%) EN 1097-6 [63]
NA, 0/4 mm	4	2510	35.02	2.44
NA, 4/8 mm	8	2450	39.88	1.6
RGA, 0/4 mm	4	2330	41.28	1.2
RGA, 4/8 mm	8	2400	42.49	1.4
RBA, 0/4 mm	4	2020	63.12	4.62
GBA, 0/2 mm	2	2440	78.15	2.21
RTA, 0/2 mm	2	1540	63.13	2.24

**Table 2:** Cementitious composite characteristics.

Sample Code	Aggregate	NT Content (%WT)	Density (kg/m^3^) EN 1015-10 [79]	Water Absorption Coeff. (%) EN 1015-18 [80]	Compressive Strength (N/mm^2^) EN 1015-11 [81]
R1-0NT	NA	0	2298	0.16	59.18
R1-3NT		3	2453	0.09	60.73
R1-5NT		5	2405	0.03	59.04
R2-0NT	NA partially substituted with RGA	0	2262	0.09	56.93
R2-3NT		3	2287	0.08	57.47
R2-5NT		5	2307	0.07	56.47
R3-0NT	NA partially substituted with RBA	0	1993	0.27	57.75
R3-3NT		3	2111	0.12	58.87
R3-5NT		5	2095	0.09	58.23
R4-0NT	NA partially substituted with GBA	0	2246	0.08	68.96
R4-3NT		3	2331	0.03	69.55
R4-5NT		5	2334	0.04	69.88
R5-0NT	NA partially substituted with RTA	0	2054	0.37	37.58
R5-3NT		3	2083	0.16	43.68
R5-5NT		5	2077	0.11	35.19

**Table 3:** Kinetic indicators of linear polarization.

Sample Code	E (i = 0) [mV]	R_p_ [kohm cm^2^]	i_corr_ [μA/cm^2^]	β_a_ [mV]	β_c_ [mV]	v_corr_ [μm/an]
R1-0NT	−867.9	2.29	11.2281	229.3	−130.5	131.3
R1-3NT	−751.6	5.34	5.2873	593	−113.2	61.84
R1-5NT	−556.6	20.44	0.6444	124.6	−54.1	7.537
R2-0NT	−347.2	31.91	0.8605	213.4	−135.7	10.06
R2-3NT	−290.3	68.68	0.4741	366.6	−140.6	5.545
R2-5NT	−457.2	48.79	0.5366	184.8	−145.7	6.276
R3-0NT	−457.3	21.2	1.2836	174.2	−161.2	15.01
R3-3NT	−292.5	149.85	0.1759	234.6	−116.5	2.057
R3-5NT	−232.2	391.44	0.615759	237.5	−95.8	0.7202
R4-0NT	−762.6	4.66	5.8912	479.3	-110	68.9
R4-3NT	−232.4	143.59	0.1646	281.2	−96.3	1.925
R4-5NT	−404.2	11.28	2.3995	191.8	−149.3	28.06
R5-0NT	−374.8	95.46	0.2795	262	−125.4	3.268
R5-3NT	−801.5	2.28	12.1256	422.6	−120.7	141.8
R5-5NT	−709	7.99	3.7489	654.5	−105.9	43.84

E—corrosion current, R_p_—linear polarization resistance, i_corr_—current density, β_a_—anodic slope, β_c_—cathodic slope, v_corr_—corrosion rate.

**Table 4:** Circuit element values corresponding to the Nyquist diagrams for the samples studied.

Sample Code	R_s_ (kΩ)	CPE-T (mF)	CPE-P	R_p_ (kΩ)	R_ox_ (kΩ)	CPE_ox_-T (µF)	CPE_ox_-P
R1-0NT	0.30	19.6	0.6899	2.5	1.5	70	0.0839
R1-3NT	0.25	12.5	0.7399	9.9	2.1	70	0.0839
R1-5NT	1.05	19.9	0.6699	2.5	1.9	70	0.0839
R2-0NT	1.60	8.1	0.6799	48	1.7	70	0.0839
R2-3NT	0.80	9.1	0.7129	120	1.7	70	0.0839
R2-5NT	0.25	15.5	0.7599	9.9	2.1	70	0.0839
R3-0NT	2.60	8.1	0.6299	9.6	1.9	70	0.0839
R3-3NT	1.60	9.1	0.8399	48	1.7	70	0.0839
R3-5NT	3.80	10.1	0.8399	30	1.7	70	0.0839
R4-0NT	0.50	13.6	0.6999	15	1.5	70	0.0839
R4-3NT	1.0	12.6	0.7999	25	1.7	70	0.0839
R4-5NT	1.2	11.6	0.7199	6.5	2.8	70	0.0839
R5-0NT	0.02	12.6	0.7799	48	1.5	70	0.0839
R5-3NT	0.02	14.6	0.6899	6.0	1.2	70	0.0839
R5-5NT	0.18	17.6	0.6889	6.0	1.2	70	0.0839

R_s_—solution polarization resistance, R_p_—polarization resistance of the armature, R_ox_—oxide layer polarization resistance CPE-T—dimension of the capacitor corresponding to the armature, CPE-P—characterization factor for the CPE, CPE_ox_-T—dimension of the oxide layer formed, CPE_ox_-P—characterization factor for the CPE_ox._

## Data Availability

Data are contained within the article.
